# Stress-Buffering Factors of Social Integration on Depressive Symptoms Over Time in Late-Life

**DOI:** 10.1093/geroni/igab046.3333

**Published:** 2021-12-17

**Authors:** Emily Kinkade, Heather Fuller

**Affiliations:** 1 North Dakota State University, Fargo, North Dakota, United States; 2 North Dakota State university, Fargo, North Dakota, United States

## Abstract

The negative impacts of stress on older adults’ well-being are well documented, and social integration is posited as protective against such detrimental effects. Previous research illustrates the stress-buffering effect of social relationships on both physical and mental health, such as depressive symptoms, in older adults. The purpose of this study was to expand on prior findings by investigating the longitudinal stress-buffering effect of various dimensions of social integration on depressive symptoms among an older sample. Four waves of data were drawn from the Social Integration and Aging Study, including 416 older adults (ages 60-100). Subscales of the Social Integration in Later Life Scale measuring frequency and satisfaction with social ties and community interaction were used to assess distinct dimensions of social integration. Multilevel modeling demonstrated that two facets of social integration—satisfaction with social ties and frequency of community interaction—moderated the relationship between perceived stress and trajectories of depressive symptoms over time. Participants who reported high levels of stress reported fewer depressive symptoms if they had high satisfaction with social ties and high frequency of community involvement. Interestingly, frequency of contact with social ties and satisfaction with community interaction did not similarly buffer negative effects for depressive symptoms. These findings indicate the value of remaining actively engaged in the community and maintaining meaningful relationships as older adults age. Future research should investigate programs to foster relationships and engagement between older adults and their communities, with particular consideration of populations at a greater risk for isolation.

